# Philadelphia Hospital

**Published:** 1844-04-06

**Authors:** 


### CLINICAL LECTURES AND REPORTS.

### PHILADELPHIA HOSPITAL.

### CLINIC OF THE JEFFERSON MEDICAL COLLEGE.

*February* 17, 1844.

BY PROFESSOR DUNGLISON.

[Reported by Samuel G. White.]

#### HYDROPIC CACHEXIA.

The lecture was commenced by introducing the case of hydropic cachexia presented at the last lecture, to report its progress towards a cure. The patient has nearly recovered, there being only a slight remnant of the cellular infiltration, as indicated by œdema of the ankles.

#### CARDIAC DISEASE.

The attention of the class was next directed to the case of cardiac disease, which, in consequence of the difficulty in forming a positive diagnosis, had excited so much interest on a previous occasion. (Examiner, for Feb. 10, p. 28.) The patient has become much thinner, and suffers from great dyspnœa, amounting to orthopnœa. This seems to be owing to the gravitation of the tumour on the left side of the sternum, which has increased considerably since the last lecture on the case. It has not pointed, but has expanded greatly; nor is there any manifest thinness of the integuments. In consequence of this appearance, together with the fact, that the tumour is below the integuments—for it may be felt beneath on raising the skin, and not, as in common abscess, condensed into the parietes of the sac—the Professor considers the idea of the tumour being produced by a partial aneurism of the heart, more probable than ever. Being a case so rare and interesting, the class were severally permitted to examine it; the Professor pointing out the peculiarities, and especially the heaving expansive movement, which could be felt over every part of the tumour, and which conveyed to the finger the sensation, that the pulsation was produced by a fluid driven into the sac. The true nature of the case will probably soon be revealed by death, but at present it is enveloped in much obscurity.

#### SCROFULOSIS.

The Professor then proceeded to offer some remarks on the morbid condition known as scrofula, in regard to which many indulge vague or confused views. The pathology of scrofula, and the definition of the term are differently expressed by different authors. Some define it to consist essentially of inflammation of the lymphatic glands, especially of those of the neck. This is too incomprehensive, and expresses but imperfectly the great characteristics of the disease. Although inflammation of the glands, and chiefly of the cervical, is very common in this condition, it is often absent—and it is only to be regarded as a functional expression of the morbid diathesis. The disease frequently expresses itself elsewhere, as in coxalgia, white swelling, caries, &c., and if we were guided by the above definition, we should be constantly in error. The professor believes, that scrofula is a morbid condition of the entire system of nutrition, and that, therefore, it should be classed among the cachexiæ; and that the inflammation and enlargement of the glands, like the eruption in variola, rubeola, &c., are merely attendant phenomena. The lymphatic ganglia are liable to simple inflammation from trifling irritation. A thorn, run into the finger, developes enlargement of the glands of the axilla; inflammation of the mucous membrane of the intestines gives rise to mesenteric ganglionitis, owing to the extension of the irritation to the lymphatic glands, situate between the seat of irritation and the thoracic duct, &c. In the same manner, nutritive irritation, occurring in one of a scrofulous diathesis, may occasion inflammation of the same glands. But being general in its influence, scrofulosis may express itself in various other forms beside ganglionitis. With some it has been a question, whether it be not dependent on a morbid state of the blood? The Professor believes not; because the disease, or a tendency to it, exists prior to the formation of blood. An impulse to it must exist in the materials furnished by one or both parents at a fecundating copulation; which impulse persists, and is liable to be developed at some after period of the individual's existence. This tendency may not be communicated by the immediate parents, but may descend from the grandfather or grandmother;—thus, like facial resemblances, passing one generation. Some writers, among whom is Dr. Stokes, place this affection in the system of white vessels; in which, according to them, the lymphatics execute the function of veins. This view, in the Professor's opinion, is untenable, as it implies a something which probably has no existence—white blood. He considers the want of colour in certain of the tissues, as in the conjunctiva and serous membranes, to be due to the thin stratum of red blood circulating in their vessels, which renders the colour inappreciable,—not because they are nourished by white blood. A very thin stratum of any coloured liquid, viewed by transmitted light, appears to be colourless; but if the quantity of liquid be increased, the colour is manifest. Now, this is exactly the change which occurs in inflammation of the white tissues: the quantity of red blood in the vessels is increased sufficiently to render its colour perceptible;—not, that there is an enlargement of arteries destined in health to convey white blood, so as to admit red globules, as supposed by some. There are, in fact, no white arteries, and, consequently, no white veins. If such vessels indeed existed, they would be liable to continual obstruction from the red corpuscles. The function, executed by the lymphatics, would seem to be that of breaking down the old tissues, and converting them into lymph—a fluid essentially the same in every part of the lymphatic system—which eventually enters the circulation. In other words, they are engaged in the process of assimilation, or in that of the decomposition and renovation of the tissues. From what has been said, then, scrofulosis must be considered as a peculiar morbid condition of the whole system of nutrition. It is, for the most part, a disease strictly of childhood, being frequently arrested by the universal change which occurs at the age of puberty. Yet, although usually confined to childhood, it is not unfrequently developed in the adult—and even in advanced life.

Scrofulosis is very analogous to, although not identical with, tuberculosis. They both occur in persons of the same habit and appearance; but there is this difference, that tuberculosis is most frequently developed after puberty—the period when the tendency to scrofulosis is usually diminished or arrested. A person, however, having scrofula in early life, is more liable to tuberculosis at an after period. Persons having light hair, light eyes, fair skin, rather comely in appearance, and distinguished for precocity of genius, were supposed by the old writers to be more frequently the subjects of scrofula. The Professor's own experience, however, leads him to conclude that this cachexia occurs as frequently in those of an opposite cast. The negroes of the South are very subject to it, and in its most severe forms.

When a close investigation is made, it will be found, that infants of the scrofulous habit display evidences of imperfect formation; that their development resembles, in some respects, that of the fœtus before the seventh month of utero-gestation: the abdomen is prominent; the liver large; the head large; the extremities puny, &c.;—circumstances which confirm the view, that it is a disease of the system of nutrition.

The causes of scrofula are predisposing and exciting. Although a predisposition or tendency generally exists, yet under peculiar circumstances, which tend to deprave the nutritive action of the system—to lessen its powers, and impair its healthy functions, the cachexia may be developed when no predisposition is present; and, on the other hand, if the exciting causes be avoided, a person having a scrofulous tendency may delay or effectually prevent its development, even for life. The exciting causes are numerous. Any thing that will vitiate the system, as bad air, exclusion of light, improper or insufficient food, want of exercise, the congregation of numerous persons in a confined space, &c., may excite it. When exposed to like influences, all animals become diseased; hence, there is rot among sheep, measles among swine, pip amongst fowls, &c., &c., when closely congregated together. Such being the circumstances favourable to its development in man, it is most prevalent where these circumstances obtain. Hence it prevails extensively in factories, narrow, crowded streets, eleemosynary institutions for children, &c. &c.

As illustrative of the several points of the lecture, the Professor now introduced various scrofulous patients. Two of the number only exhibited evidences of inflammation of the cervical glands; one of whom presented a specimen of the unseemly scars produced by the spontaneous rupture of the ulcerated glands. Several others were affected with coxalgia, caries of the vertebræ, &c. One was a female, labouring under a total loss of intellect, or dementia.

[This led the Professor to make some remarks on the classification of the different forms of insanity, suggested by a letter which he had received that morning from a philanthropic merchant of Boston, and which he read to the class, proposing a plan for the reciprocation of documents from the various insane establishments of this country and of Europe. The lecturer thought it important that some divisions of insanity should be determined upon; and stated, that although objections might be made to the division into *mania*, *melancholia*, *moral insanity*, *dementia*, and *idiocy*, it was probably as good as any that had been proposed.]

The observations—the lecturer remarked—which he had made concerning the nature and causes of scrofulosis, indicate that the treatment must be both hygienical and therapeutical. He considered, that even more weight should be attached to the former than to the latter. As there is defective formation, any active depletory treatment would seem to be contra-indicated, unless some peculiarity in the case should demand it. Among the therapeutical means, the class of remedies described by him as eutrophics—that is, articles which, by being received into the blood, alter its condition, and, through it, modify nutrition without necessarily increasing the secretions—have been most employed. Iodine, and its various preparations, have been very extensively administered; Lugol's solution more, perhaps, than any other, in consequence of the strong assertions in its favour by its proposer. The iodide of potassium has, also, an exalted reputation. Mercury, too, has been given, but it is often injurious, when pushed too far, in consequence of the debility it produces. Its combinations with iodine are often administered, when mercury is thought necessary. Although most flattering accounts are givenof the benefits of these different articles, the experience of the Professor does not justify him in declaring that he has found much good from them, except in rare cases. Recently, cod-liver oil—which is found to contain a small quantity of the iodide—has been highly lauded; but it is excessively disagreeable, and probably not more effective than so much olive oil, containing iodide of potassium, would be. It acts not only by the salt of iodine which it contains, but by the modified chyle which is produced by the digestion of the oil.

But hygienical measures must constitute the chief basis of general treatment. The patients should be removed from the exciting causes; the baneful influence, whatever it may be, should be taken away. Change of residence; removal to the sea shore; country air; free exercise in the open air; good and nutritious diet;—in fine, change of the entire circumstances of the individual should be inculcated. By this means, benefit will often result, the morbid tendency being arrested or greatly ameliorated. In addition to these general modes of treatment, the *expressions* of the disease demand attention. When the cervical glands become enlarged, suppurate, and are felt fluctuating, lay them open by an incision coincident with the folds of the neck, so as to prevent the unseemly scars, which invariably result when they are permitted to discharge spontaneously, or aided by poultices. The other affections, as coxalgia, caries, conjunctivitis, &c., require the treatment adapted for those diseases.

